# An Overview of an Undergraduate Diversity MCH Pipeline Training Program: USF’s Train-A-Bull

**DOI:** 10.1007/s10995-021-03332-y

**Published:** 2022-01-03

**Authors:** Anna Torrens Armstrong, Charlotte A. Noble, Juliana Azeredo, Ellen Daley, Roneé E. Wilson, Cheryl Vamos

**Affiliations:** 1 College of Public Health, University of South Florida, 13201 Bruce B. Downs Blvd., MDC 56, Tampa, FL 33612 USA; 2 University of North Texas Health Science Center School of Public Health, 3500 Camp Bowie Field, Fort Worth, TX 76104 USA

**Keywords:** Pipeline, Maternal and child health, Underrepresented minorities, Mentorship

## Abstract

**Purpose:**

To describe an undergraduate pipeline training program (PTP) designed to guide underrepresented minorities (URM) trainees into MCH-related health professions, ultimately contributing to a diverse maternal and child health (MCH) workforce that can improve health outcomes for all women/mothers, children, and their families, including fathers and children with special healthcare needs.

**Description:**

Three cohorts with 35 total undergraduate trainees were recruited to participated in the 2 years USF MCH PTP program where they were mentored, trained, guided, and supported by program faculty/staff. Students were recruited early in their education track, and the program was individually tailored based on trainees’ educational discovery stages. Key program components included seminars, summer institutes, public health courses, mentorship, internship, experiential learning opportunities, and professional networking opportunities.

**Assessment:**

The majority of the undergraduate participants were diverse URMs including Hispanic/Latino (37.1%), Black/African American (31.4%), Asian (20%), and American Indian/Alaskan Native (5.7%) trainees. Out of all the cohorts, 51.4% were first-generation college students and 74.3% had economic hardships (i.e., PELL Grant, FAFSA). Resulting from the program, all cohorts increased in educational discovery stages, one-third enrolled in health-related graduate studies and half joined the MCH workforce.

**Conclusion:**

Recruitment in pipeline programs should be intentional and meet students where they are in their education discovery stage. The use of educational discovery stages within a pipeline program are useful in both tailoring curriculum to individuals’ needs and assessment of progression in career decision-making. Mentoring from program staff remains an important component for pipeline programs.

## Significance

The dissemination of the outcomes from MCH diversity pipeline training programs contribute to the knowledge and evidence base of best practices for future programs to consider. Educational pipelines in MCH are acknowledged as an essential part of building a diverse future MCH workforce, but little is known about the various programs that have been implemented.

This article highlights the use of the educational stages of discovery within an MCH diversity pipeline program as both a method of assessment and tailoring of program components.

## Introduction

With the challenges of the evolving healthcare system and the urgent need to address health disparities and inequalities through a social determinants of health lens in Florida and the nation, we must ensure availability of a competent, passionate, and reflective MCH workforce to improve health outcomes for MCH populations. High-quality MCH training programs that recruit, mentor, and engage the future MCH workforce are critical to achieving this goal. This future workforce must reflect members of historically underrepresented minorities, in the quest of reducing health disparities in MCH. An AHRQ report (2018) reinforces the need for a diverse workforce to provide high-quality, culturally and linguistically responsive care to MCH populations. Diversity among healthcare providers improves patient access, satisfaction, and quality of care (Moy & Freeman, 2014).

With health workforce shortages, increased attention on recruiting and training a diverse workforce is imperative (Duffus et al., 2014). The U.S. workforce among 30 health occupations consists of a white majority (64.4%), followed by: Hispanics (16.1%); Black/African Americans (11.6%); Asians (5.3%); American Indians/Alaskan Natives; (0.6%) and Native Hawaiians/Other Pacific Islanders (0.2%) (HRSA, 2015). Among the 47,000 public health professionals who participated in the public health workforce interests and needs survey, few are Black/African American (16%), Hispanic/Latino (14%), or Asian (5%) (de Beaumont Foundation, 2017). Florida also lacks a diverse workforce and faces widening cultural gaps, as Hispanics and African Americans comprise almost 25% and 17% of the population, but only 17.6 and 5.5% of licensed physicians, respectively (AHRQ, 2018; Desantis & Rivkees, 2020; Florida Department of Health, 2016; U.S. Census Bureau, 2016).

Pipeline programs have shown to increase diversity across healthcare; thus, the dissemination of program strategies and outcomes are important (Kuo et al., 2015). Given that MCH pipelines are developed based on overarching proposal guidelines, and result in unique, contextualized approaches, there is added value in disseminating program overviews. Additionally, there are very few publications providing such description (Kuo et al., 2015). By sharing these programs, we can move towards a common model of the critical elements that should be included in all MCH pipeline programs of the future (Petersen, 2019). Our program presents a student-centered approach that attempts to lower barriers for a population of students that have multiple responsibilities, allowing the program to meet their needs to maximize their level of engagement. This paper describes a pipeline training program (PTP) designed to guide URM trainees into MCH-related health professions. We also include a brief overview of the methods used to evaluate the program emphasizing the outputs, initial and intermediate outcomes based on the program logic model (Table 1), along with challenges and lessons learned.

**Table 1:**
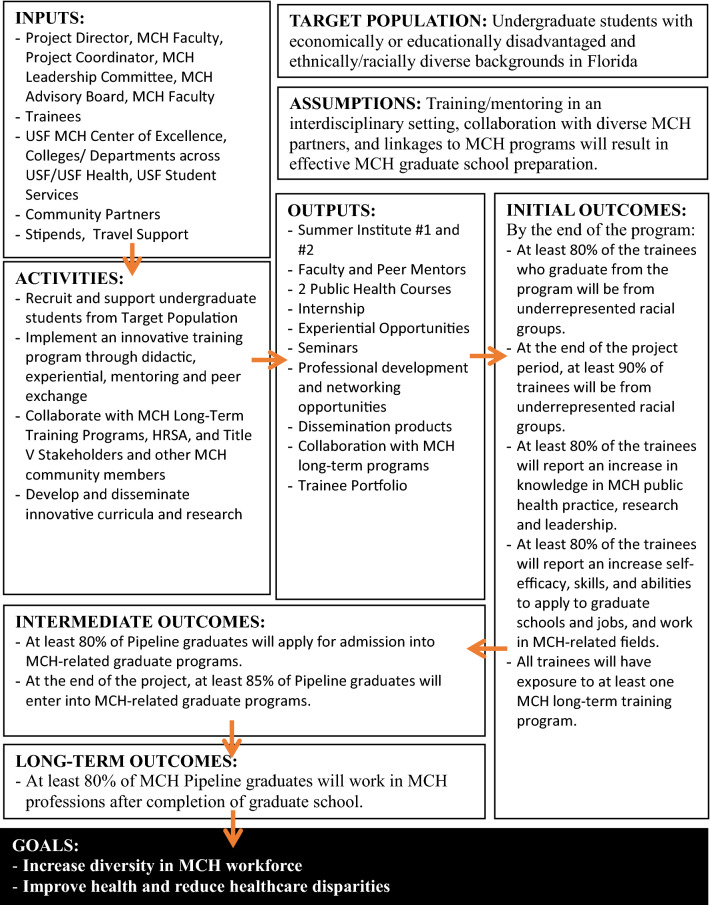
USF MCH PTP logic model

## Description

To address the need for a highly skilled and diverse MCH workforce, the University of South Florida (USF) MCH PTP aimed to recruit, train, mentor, and provide enriching experiences with the goal of guiding URM undergraduate trainees into MCH-related health professions to improve levels of representation, reduce health disparities, and increase access to health care for vulnerable, underserved populations.

This PTP had four overarching goals: (1) recruit and support cohorts of undergraduate students from economically and educationally disadvantaged and racially or ethnically diverse backgrounds into a high-quality training program that prepares them for a successful trajectory into MCH graduate training and professions; (2) implement an innovative training program through didactic, experiential, mentoring and peer exchange modalities that expose students to MCH leadership, broad public health perspectives, interdisciplinary training and practice, and cultural and linguistic competencies; (3) collaborate broadly with MCH Long-Term Training Programs, HRSA and Title V stakeholders and other MCH community members; and (4) develop and disseminate innovative curricula and research to advance MCH training and practice.

Program components were developed within the framework of the stages of educational discovery to maximize program success (Arnold et al., 2015). These stages include: (1) *decider:* expresses interest in health science; exploring degree options; (2) *explorer:* declared health science; exploring double majors/minors and discussing links with coursework and other opportunities; (3) *maximizer:* in middle of program; seeking practice opportunities; and (4) *mover:* approaching graduation; preparing for post-graduate training or workforce (Arnold et al., 2015). Trainee stage data was assessed throughout the program for evaluation purposes. Program staff formed strong connections with trainees and engagement continues post-program, supporting ongoing insights into their educational and professional trajectories.

We recruited three cohorts of 12 trainees into the MCH PTP. Support for trainees included stipends (provided after completing summer institutes) and tuition waivers for required coursework.

### Recruitment

A dynamic, student-centered (i.e., focused on student needs and involvement rather than program staff expectations) plan was developed using a combination of informal and formal recruitment avenues. The two-tiered approach included: (1) recruitment from within identified feeder programs (e.g., student support services, program advisors, one-on-one recruitment at events, program announcement to instructors and program administrators); and (2) recruitment from additional informal and formal methods and strategies (e.g., online university platforms, social media, career fairs, meet and greets).

Recruitment strategies were implemented during Spring semester and focused on undergraduate students in any major in the second half of their freshmen (first) year and/or beginning of their sophomore (second) year. Trainees participated in the program for two years starting in the Spring semester and completed the program in the first half of their fourth year. Completing the program in their fourth year was optimal in the context of the educational discovery stages as it allowed mentorship and guidance in the graduate school application process. Eligibility criteria included: current undergraduate enrollment (full-time) at USF; U.S. citizenship or permanent resident visa; demonstrate economically or educationally disadvantaged status (e.g., financial aid eligible); first generation student; family hardship (e.g., foster family); descendants of immigrant or migrant family or multi-language proficiency; self-identify as URM; and a demonstrated interest in MCH and/or pursuing graduate training.

### Program Components

The USF MCH PTP curriculum had six components: (1) seminars; (2) summer institutes; (3) public health courses; (4) mentorship; (5) internship; and (6) professional networking opportunities (see Table 2). The first year of the program emphasized MCH knowledge through coursework, seminars and the summer institute. During the second year, seminar and summer institute topics focused on graduate school preparation and practical application through the development of a research proposal poster. This scaffolded approach allowed for distributed practice of content and refinement of program components based on trainee educational discovery stage.

**Table 2:** Program timeline and components

Academic year	Sophomore			Junior				Senior	
Months	January–June		July–December		January–June		July–December		
Semester	Spring	Summer		Fall	Spring	Summer		Fall	
1. Public health courses				Foundations of MCH (HSC 4579)	Intro to health disparities and social determinants of health (PHC 4933)				
2. Summer institute		Summer institute #1				Summer institute #2			
3. Seminars	4 Seminars	1 Seminar		4 Seminars	4 Seminars	1 Seminar		4 Seminars	
4. Mentorship	Mentors assigned	Mentorship throughout program							
5. Internship				Internship (clinical shadowing or field experience)					
6. Professional networking opportunities				MCH-long term collaborat-ive meetings	USF health research day	Florida public health association			
*Additional activities*									
Graduate school application						Apply to MCH-related health fields graduate school			
Evaluation									

### Seminars

The program commenced with monthly seminars to foster key comprehension and application of relevant MCH topics. The seminars, hosted and facilitated by program faculty and staff, included guest lectures or discussions from other diverse health professional faculty and MCH community leaders and professionals (e.g., Florida Perinatal Quality Collaborative, Florida Covering Kids and Families, USF Student Affairs, USF Writing Studio). Some seminars were also co-hosted with USF’s Center of Excellence in MCH encouraging a continuous trajectory of learning and mentoring. Example seminar topics included: program orientation; MCH 101 part 1 and part 2; community-based research in infant and child health; quality improvement in MCH; graduate/doctoral program student panel; professionalism; social networking; systems approach; MCH advocacy and policy; emotional intelligence; and communication/health literacy.

### Summer Institutes

Trainees participated in two virtual, self-guided institutes delivered as modules (five per summer) via the Canvas Learning Management System. The first institute introduced trainees to the field of MCH, research skills, and internship opportunities. The topics by module included: Research 101 Part 1; research 101 Part 2; research ethics; interdisciplinary practice; and conflict resolution and negotiation. The first summer institute included activities such as discussion boards, CITI training, and research activities. A more detailed description is provided later. The second institute expanded trainees’ professional development skills with an emphasis on graduate school programs and applications. Module topics included: family-centered care; cultural and linguistic competency; resumes, CVs, and finding your dream job; and personal statements, cover letters, and finding the right graduate school. The second summer institute included case studies with open discussion boards, resume, personal statement and cover letter peer review sessions, and guided activities on conducting research to find dream jobs and graduate programs. All modules contained pre and post-tests to assess changes in knowledge.

### Public Health Courses

Trainees were required to take two undergraduate public health courses during the program, Foundations of Maternal and Child Health and Introduction to Health Disparities and Social Determinants of Health. The first course, Foundations of MCH, served as an introductory course, providing an overview of MCH issues and trends. The objectives of this course are organized around the knowledge of health assessment and interventions for families and children. The second course, Introduction to Health Disparities, uses a social ecological framework to provide a broad overview of health disparities in the U.S. and multi-level factors influencing those disparities. It also examines social and cultural determinants of health including race/ethnicity, geography, socioeconomic position, gender, sexual orientation, disability status, migration status, age, religion and spirituality. If a trainee had already taken one of these courses, tuition was offered for another public health elective from a comprehensive list including, but not limited to: Women’s Health; Nutrition and Disease; or Public Health Education Theory and Behavior. For flexibility, courses could be taken at any time in the program.

### Mentorship

The MCH PTP provided trainees with mentorship from multiple stakeholders: (1) program faculty and staff; (2) graduate students in health professions; and (3) USF and MCH faculty. Program staff mentored trainees in navigating life challenges often faced by URM students, while faculty offered additional support and guidance on succeeding academically, refining research interests, and planning MCH careers. Program faculty and staff assisted trainees in aligning their academic, professional and career expectations; promoting professional development and developing research skills; building leadership and self-advocacy skills; and guiding graduate program or workforce preparation activities. Program faculty and staff matched a diverse pool of graduate health student mentors with trainees to provide student perspectives on navigating graduate school preparation, and guidance on MCH research poster proposals that were presented in the second year of the program at the Annual Chiles Center Symposium. An expected outcome from this mentorship structure included trainees accompanying their mentors to faculty research team meetings to increase additional research exposure and opportunities.

### Internship

After completion of summer institute 1, trainees were supported in identifying and completing a clinical or practice track internship. Both options were considered, as trainees represented a variety of health disciplines with different career paths. Internships made available to trainees included the following: Champions for Children [a local MCH organization focusing on community-based programming such as the A Breastfeeding & Childbirth (ABC) Program, Baby Bungalow Program, Family Learning Center, Healthy Families, and Parents as Teachers]; Shriners Hospital for Children, Advent Health and community physician shadowing (all with a focus on clinical settings). Trainees were supported in applying to Title V Internships, providing a unique opportunity to work in a state MCH agency. There were five trainees accepted into Title V internships with placements in the following locations: the Tennessee Department of Health, the Health Policy Institute of Ohio, the Public Health Department of Georgia State, and the North Carolina Department of Health and Human Services. These trainees participated in a variety of research activities including: participating in strategic planning, conducting literature reviews, conducting interviews and focus groups, developing survey questions and presenting findings.

### Professional Networking Opportunities

Trainees attended the USF Health Research Day event during their first year, guided by a graduate student. USF Health Research Day aims to encourage interdisciplinary research among undergraduate and graduate students guided by faculty, staff and keynote speakers. This event features more than 300 poster presentations with about 80 judges who nominate poster authors for research awards. For most trainees, this event was their first exposure to MCH-related research and provided a platform to network with other students, faculty, and health professionals. Additionally, trainees were encouraged to attend other local or regional conferences. There was a strong emphasis on attending Making Lifelong Connections which focused on leading, networking, and career development of current and former MCHB-funded trainees. The Florida Public Health Association conference supports Florida’s public health professionals and students in professional development, dissemination and networking. Trainees were invited to attend the Florida Family Leaders’ Summit, which brings various stakeholders together to learn about how to support family engagement for children and youth with special healthcare needs. Other shared opportunities to encourage professional development included Activist Lab events, scholarship opportunities, student organization engagement opportunities, National Women’s Health Week seminars, USF Diversity Committee events, and local research and job opportunities.

## Methods

This MCH PTP assumed that training and mentoring in an interdisciplinary setting, collaboration with diverse MCH partners, and linkages to MCH programs will result in effective MCH graduate school preparation. The stakeholders (e.g., project director, project coordinator, MCH faculty, community partners) and other inputs (e.g., stipends, tuition) led to the development of a series of program components and an evaluation plan to meet initial and intermediate outcomes (Table 1). Trainees’ demographic data were collected during the application and acceptance process. Pre- and post-surveys were administered during seminars and summer institute 1 to identify changes in MCH knowledge and MCH research knowledge.

Trainees completed pre- and post- self-efficacy surveys at the start and end of the program. This survey consisted of 15 questions (five point-Likert scale, Strongly Agree-Strongly Disagree) totaling a possible score of 75. The questions focused on long-term career goals, MCH employment/research/funding opportunities, and the graduate school application process. Program staff formed interpersonal relationships with trainees throughout their time in the program, facilitating the collection of long-term data (i.e., admission into graduate programs or workforce). Ongoing follow-up is conducted via email every 6 months to update trainee information and relevant long-term outcomes such as graduate school application, admission or work in MCH.

Although trainee educational discovery stage progression was not listed as an outcome in the logic model, it was used in designing program components. We collected this data at three points in the program: start, mid-point, and end. Trainees learned about educational discovery stages at program orientation and completed their first self-assessment. During one–one–one meetings with program staff, trainees re-assessed their stage. The data presented in this manuscript were collected as part of program evaluation and was considered exempt from IRB review and does not include clinical or patient data.

### Outcomes

By Fall 2020, 35 trainees have completed the MCH PTP. Program staff communicated with trainees during and after the program to collect data (e.g., student/employment status, e-mail address, five to 10-year goals, social media). One trainee was asked to leave the program for not fulfilling agreed upon responsibilities. Trainees identified as mostly female (91%; 9% male). A few trainees (11.4%) graduated earlier than planned and thus exited the program early. Five trainees were accepted into Title V internships. Positive results across the program presented elsewhere included increases in MCH knowledge, MCH research knowledge and self-efficacy.

#### Trainee Diversity

Recruitment of participants was consistent with the diversity goals of this program. Trainees were asked to self-identify race/ethnicity and first-generation status or economic hardship or both (e.g., PELL Grant) (Table 3).

**Table 3:** Trainee demographics and educational/economic status

	Number of trainees	Percentage of trainees (%)
Race/ethnicity		
Hispanic/Latino(a)^a^	13	37.1
White/Caucasian	11	31.4
Black/African American	11	31.4
Asian	7	20.0
American Indian/Alaskan Native	2	5.7
Other	2	5.7
Educational/economic status		
First-generation student	18	51.4
Economic hardship	26	74.3

^a^Some trainees were Hispanic/Latino ethnicity with different race so total exceeds 35

#### MCH Knowledge and MCH Research Knowledge

Paired samples t-tests were used to assess changes in pre- and post-mean scores for changes in MCH knowledge and research knowledge within each cohort, while ANOVAs were used to compare across cohorts. MCH knowledge increased within each cohort across all topics: MCH 101 part 1 (topics included foundations of MCH, overview of MCH populations and indicators, health disparities); MCH 101 part 2 (topics included overview of MCH theoretical frameworks, social determinants of health, socioecological model, lifecourse and systems theory); health literacy (topics included significance, at-risk populations, role in policy, research and practice); and quality improvement (topics included role in improving outcomes, Plan-Do-Study-Act cycle, implementation barriers and success) (Table 4). While the differences observed were significant (p < 0.05), this must be interpreted carefully due to the small sample size. There were no significant differences observed across cohorts.

**Table 4:** Average pre-post change of MCH knowledge and MCH research knowledge

	Cohort 1	Cohort 2	Cohort 3
MCH knowledge			
MCH 101 Pt 1	9.33* (0–18)	6.90* (2–13)	6.67* (− 1 to 12)
MCH 101 Pt 2	13.42* (3–27)	17.0* (5–30)	11.73* (5–19)
Health literacy	6.27* (1–11)	9.10* (4–15)	N/A**
Quality improvement	6.90* (4–9)	6.82* (4–9)	5.45* (2–8)
MCH research knowledge			
Research 101 Pt 1	11.42* (7–20)	9.58* (0–17)	10.58* (0–20)
Research 101 Pt 2	8.17* (3–12)	7.25* (3–11)	8.82* (6–14)
Research ethics	10.25* (− 2 to 20)	8.08* (2–15)	6.83* (0–13)
Interdisciplinary practice	8.67* (− 1 to 17)	9.50* (2–17)	5.92* (0–10)
Conflict resolution	6.92* (0–16)	6.92* (0–13)	5.43 (0–14)

*p < 0.05

**Cohort 3 received an alternate topic seminar due to a scheduling conflict

MCH research knowledge increased within each cohort across all topics: Research 101 part 1 (topics included purpose, process, approaches, funding for research); Research 101 part 2 (topics included qualitative and quantitative methods, data collection and analysis, literature reviews); research ethics (topics included history of human subjects research, principles in Nuremberg code and Belmont report, CITI training); interdisciplinary practice (topics included definitions and benefits/barriers to disciplinary, multidisciplinary and interdisciplinary work, elements of effective interdisciplinary teams); and conflict resolution (define sources and types of conflict, strategies to resolve conflict, skills for successful negotiation) (Table 4). Again, we observed significant differences in MCH research knowledge across all MCH topics covered (p < 0.05; with one exception of cohort 3 showing not significant for conflict resolution). There were no significant differences observed across cohorts.

#### Self-Efficacy

Findings from the pre- and post-self-efficacy surveys suggest the MCH PTP may have had an impact on trainee self-efficacy in applying to health-related graduate studies and positions in the MCH workforce. Significant differences in self-efficacy (p < 0.05) were observed across all cohorts. Survey questions showing the highest average increase pre-and post- focused specifically on self-efficacy as it applies to identifying funding or scholarship opportunities for graduate school and research activities supporting graduate school application.

#### Trainees Post-Graduation

An expected outcome of the MCH PTP was for trainees to enroll in a health or MCH-related graduate program or enter the MCH workforce directly. The curriculum supported the graduate school application process and program staff encouraged trainees to request recommendations for their applications. Program faculty provided a total of 31 recommendations for trainees for graduate school, employment, internships, fellowships, and awards. Currently, 40% of all trainees have been admitted to graduate or professional schools, with most in a health-related area (see Table 5). The last cohort completed the program in 2020, therefore follow-up will continue.

**Table 5:** Trainee graduate program admission and workforce area post-program

Graduate program	Trainees
Masters in medical sciences (MSP3)	1
Master of public health (MPH)	2
Master of social work (MSW)	1
Doctor of nursing practice (DNP)	1
Doctor of psychology (PsyD)	1
Doctor of medicine (MD)/Doctor of osteopathic medicine (DO)	5
Master of health administration (MHA)	1
Other [Accelerated second nursing degree; master in geopolitics and strategic studies; master of business administration (MBA)]	2
Total	14 (40%)

Area of employment in health field	Trainees
Nursing/clinical	8
Public health	7
Research	1
Social work	2
Total	18 (51.4%)

Approximately half of trainees are in MCH-related workforce positions (Table 5). The remaining trainees are in various stages of completing their undergraduate degrees or applying to graduate programs.

#### Educational Discovery Stages

A positive average increase in educational discovery stage progression was shown among all three cohorts (Table 6). A change of one or greater indicates a transition to the next stage (e.g., decider to explorer; maximizer to mover). Some trainees fell between two discovery stages. While this outcome demonstrates trainees transitioned to subsequent stages, several limitations should be taken into consideration including sample size, and threats to internal validity such as history and maturation.

**Table 6:** Changes in educational discovery stages

Cohorts	Participants	Average % change^a^
Cohort 1	11 trainees	+ 1.25
Cohort 2	12 trainees	+ 1.00
Cohort 3	12 trainees	+ 1.29

^a^A change of one or greater indicates a transition to the next stage

## Discussion

A student-centered recruitment plan led to a group of diverse undergraduate trainees with an interest in the field of MCH. The comprehensive, scaffolded program using complimentary curriculum components exposed trainees to relevant MCH knowledge, content and career opportunities. Mentorship on various levels paired with support and training in the graduate school application process resulted in success among trainees in their application and admission to a variety of health-related and MCH graduate programs. Engaging in the development of a research proposal and the experience of presenting research provided the opportunity for application of content, and a competitive advantage to graduate school applications.

The use of educational discovery stages is an effective approach to raise awareness and build interest in MCH careers among URM students. Repeated assessment of trainee educational discovery stage allowed program staff to refine program components to meet trainee needs, such as, identifying relevant and appropriate internship experiences. For example, several students who presented as “maximizer” (e.g., seeking practice opportunities) were encouraged to apply for the Title V internship program.

### Challenges

We encountered multiple challenges throughout program implementation. As a new initiative, raising awareness of a program was labor intensive; however, recruitment self-perpetuated after year one. Trainees reported a perceived overlap in content between the didactic program components. To prevent this redundancy, minor adaptations were made (e.g., excluded topics from seminars and summer institutes).

Retention issues arose primarily due to unanticipated early graduation (n = 5) based on a university-system policy related to excess credit hours, with only minimal program dropout (n = 1). Several strategies were added to address program retention, including a memorandum of understanding, ongoing mentorship, and frequent touch points. Additionally, trainee schedules were assessed each semester to address needs and identify barriers to participation. Maintaining contact with trainees’ post-program is also challenging. Establishing meaningful relationships with trainees during the program and maintaining engagement post-program are key strategies for follow-up.

Delivering the program under the evolving circumstances of the COVID-19 pandemic required program staff to quickly make necessary modifications to ensure trainees received the same quality program. Given part of the program was online via Canvas, trainees were already accustomed to some online program engagement.

Due to the nature of grant-funding, a sustainability plan helps ensure continuity. A five-year grant cycle provides time for initial successes that may earn university recognition and garner support. Program modifications will likely need to be made, but sustaining key program elements is critical to build a diverse MCH workforce.

### Recommendations

Program staff used multiple methods to collect trainee feedback to identify strengths and areas of improvement including pre- and post-surveys, focus groups, and exit interviews. An emergent theme across cohorts was the presence of a consistent program coordinator who trainees identified as a mentor. Trainees reported benefiting from a program coordinator offering support for URM and first-generation students, providing a safe space for open discussion. These benefits, noted by program staff, led to an open-door policy for trainees. Future pipelines should consider this policy in their staffing plan, as it created a sense of connectedness among trainees.

Relatedly, convening a culturally congruent mentoring match with graduate students provides meaningful relationships, facilitates student success and increases the likelihood of trainee engagement and commitment to MCH careers (Wyatt & Belcher, 2019). Program staff identified specific supports for graduate student mentors. The addition of a mentor orientation paired with a small stipend resulted in positive mentor-trainee satisfaction based on the results of a post-survey for mentees. The post-mentorship evaluation included questions on the description and function of the relationship; mentorship quality; mentorship components (i.e., academic and career development, research component); and overall satisfaction. Given that this was only a post-survey, care interpreting these results must be taken.

Pipeline programs should also encourage various forms of engagement within the MCH field (e.g., Title V internships, community-engaged learning) as it has been shown to increase confidence in future career decisions (Healthy People 2030, 2020; Kolb & Fry, 1975). According to trainees, participation in these types of activities solidified their understanding of MCH. Continued use of the educational discovery stages for assessment and tailoring will build the evidence to support its use in pipeline programs.

### Limitations

Despite trainee success in a program, limitations still persist. The mentorship component was originally intended to be a triad relationship between trainee, MCH faculty, and graduate student mentor. Challenges with scheduling arose leading to adaptations to this component. Graduate students were matched as mentors to support trainees in developing their research proposal poster and program staff provided ongoing mentorship. Although a faculty member was not assigned to all trainees, they were encouraged to connect with those faculty with aligning research interests. Given the recent completion of this program, long-term outcomes are not available. Outcomes are expected to change over time based on the continued trajectory of trainees’ education and career paths.

## Conclusion

Existing health disparities and a lack of diversity in the MCH workforce continues to drive the need for a pipeline of well-trained and reflective future MCH professionals. A diverse MCH workforce supports patient access and satisfaction in addition to quality of care. Continued funding for pipeline programs is important and should look to support the dissemination of program results to improve overall outcomes and strengthen future efforts while building the evidence base. For example, dissemination should include the ascertainment of critical pipeline components that lead to success, such as mentoring and tailoring to student educational discovery stages.

Our approach to a layered, dynamic curriculum that maintained a focus on trainee educational discovery stage helped guide student success in graduate school admission. Trainees moved through the educational discovery stages within our program, demonstrating our curriculum was successful in supporting their journey while also providing a metric for change.

## Data Availability

Not applicable.

## References

[CR1] Agency for Healthcare Research and Quality. (2018). National healthcare quality and disparities report. Retrieved from https://www.ahrq.gov/research/findings/nhqrdr/nhqdr18/index.html

[CR2] Arnold LD, Embry ES, Fox C (2015). Advising undergraduate public health students: A phased approach. Public Health Reports.

[CR3] de Beaumont Foundation. (2017). Public health workforce interests and needs survey. Retrieved from https://www.debeaumont.org/phwins-findings/

[CR4] DeSantis, R., & Rivkees, S.A. (2020). Physician workforce annual report. Retrieved from http://www.floridahealth.gov/provider-and-partner-resources/community-health-workers/physician-workforce-development-and-recruitment/2020DOHPhysicianWorkforceAnnualReport-10-28-20FINAL.PDF.

[CR5] Duffus WA, Trawick CT, Moonesinghe R, Tola J, Truman BI, Dean HD (2014). Training racial and ethnic minority students for careers in public health sciences. American Journal of Preventive Medicine.

[CR6] Florida Department of Health. (2016). FloridaCHARTS: Infant deaths per 1000 live births 3-year rolling rates. Retrieved from http://www.flhealthcharts.com/ChartsReports/rdPage.aspx?rdReport=InfantDeath.DataViewer&cid=0053

[CR7] Healthy People 2030. (2020). Expand public health pipeline programs that include service or experiential learning—PHI-R02. Retrieved from https://health.gov/healthypeople/objectives-and-data/browse-objectives/public-health-infrastructure/expand-public-health-pipeline-programs-include-service-or-experiential-learning-phi-r02

[CR8] Kolb DA, Fry R, Cooper C (1975). Towards an applied theory of experiential learning. Theories of group processes.

[CR9] Kuo AA, Verdugo B, Holmes FJ, Henry FA, Vo JH, Perez VH, Inkelas M, Guerrero AD (2015). Creating an MCH pipeline for disadvantaged undergraduate students. Maternal and Child Health Journal.

[CR10] Moy E, Freeman W (2014). Federal investments to eliminate racial/ethnic health-care disparities. Public Health Reports.

[CR11] Petersen DJ (2019). Channeling our legacy into our future: The importance of the MCH pipeline training program. Maternal and Child Health Journal.

[CR12] U.S. Census Bureau. (2016). QuickFacts. Retrieved from http://quickfacts.census.gov/qfd/index.html

[CR13] U.S. HRSA Health Workforce. (2015). Sex, race, and ethnic diversity of US health occupations (2011–2015). Retrieved from https://bhw.hrsa.gov/sites/default/files/bureau-health-workforce/data-research/diversity-us-health-occupations.pdf

[CR14] Wyatt GE, Belcher HME (2019). Establishing the foundation: Culturally congruent mentoring for research scholars and faculty from underrepresented populations. American Journal of Orthopsychiatry.

